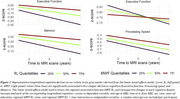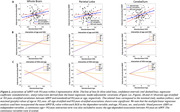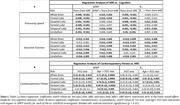# Cortical Myelination, Cognition, and Cardiovascular Fitness: New Insights using Advanced Magnetic Resonance Relaxometry

**DOI:** 10.1002/alz.093768

**Published:** 2025-01-09

**Authors:** Alex Guo, Mary E Faulkner, Zhaoyuan Gong, Jonghyun Bae, John P Laporte, Mustapha Bouhrara

**Affiliations:** ^1^ National Institute on Aging, Baltimore, MD USA; ^2^ Laboratory of Clinical Investigation, National Institute on Aging, Intramural Research Program, Baltimore, MD USA

## Abstract

**Background:**

Alterations in grey matter (GM) are known to contribute to cognitive decline in aging and dementia. Recent evidence suggests that individuals with higher cardiorespiratory fitness (CRF) may have a lower risk of GM atrophy and subsequent cognitive decline. However, the role of cortical myelination in cognitive decline and its association with CRF remains elusive. We used two microstructure MRI measures: MWF, which is a direct measure of myelin content, and R1, which is sensitive to lipids, the main constituents of myelin, to first, investigate the relationship between myelination and longitudinal changes in cognition, and second, determine whether higher CRF is associated with higher myelination in cortical regions.

**Method:**

123 participants (55.1±20.4 years) underwent our BMC‐mcDESPOT MRI protocol for MWF and R1 mapping with mean values calculated in 6 GM regions (Table 1). Cognitive performance scores were obtained retrospectively at the time of MRI and before. VO2max, the gold standard measure of CRF, was also measured using our established treadmill protocol. The association between each MRI measure and changes in cognition was evaluated using a linear mixed‐effects model. The association between VO2max and each MRI measure was assessed using a multiple linear regression model. Relevant covariates were included in both models (Fig1. caption & Fig.2 caption).

**Result:**

Lower myelin content in various GM regions, indicated by lower MWF and R1 values, is associated with a steeper decline in all cognitive domains (Fig. 1). These associations were prominent with processing speed and executive function (Table 1A). Moreover, higher VO2max was associated with higher myelin content in several GM regions (Table 1B). An interaction between age and VO2max revealed a steeper positive slope in the older age group, suggesting that the beneficial effect of VO2max on MWF is more pronounced at older ages; and a negative slope in the lower VO2max group, indicating that lower VO2max levels are associated with more rapid demyelination with advanced ages (Fig.2, Table 1B).

**Conclusion:**

These original findings highlight the importance of cortical myelination in cognitive functioning, and suggest that improving CRF could represent a non‐pharmaceutical intervention toward better myelination and offset subsequent cognitive decline.